# Divergent Embryo Responses to Chemical Cues in Two Freshwater Fishes with Different Parental Care Strategies

**DOI:** 10.3390/ani15243511

**Published:** 2025-12-05

**Authors:** Ning Zhang, Qinlei Li, Minghui Li, Chris K. Elvidge, Chuke Deng, Deshou Wang, Shijian Fu, Jigang Xia

**Affiliations:** 1 Fish Ecology and Conservation Research Center, Chongqing Normal University, Chongqing 401331, Chinashijianfu9@cqnu.edu.cn (S.F.); 2 Key Laboratory of Freshwater Fish Reproduction and Development (Ministry of Education), School of Life Sciences, Southwest University, Chongqing 400715, China; 3 Department of Biology, Carleton University, 1125 Colonel By Drive, Ottawa, ON K1S 5B6, Canada; 4 Laboratory of Evolutionary Physiology and Behavior, Animal Biology Key Laboratory of Chongqing Education Commission, Chongqing Key Laboratory of Conservation and Utilization of Freshwater Fishes, Chongqing Normal University, Chongqing 401331, China

**Keywords:** chemical communication, cognition, damage-released chemical alarm cue, companion embryo odour, innate recognition, maternal odour

## Abstract

Chemical information is one of the most important modes of communication among aquatic organisms. However, it remains unclear whether fish embryos are innately responsive to ecologically-related chemical cues from the cues of their cohort competitors, potential caring or risky cues from parents, or chemical alarm cues from damaged embryos, and whether these response patterns differ between species that model alternative parental care strategies. Here, we examined embryo responses in heart rate and incubation performance to different conspecific chemical cues (companion embryo odours, maternal odours, maternal + companion odours, embryonic alarm cues, or water controls) in zebrafish (*Danio rerio*; no parental care) and Nile tilapia (*Oreochromis niloticus*; high parental care). Zebrafish embryos did not respond to companion embryo odours but did respond similarly to maternal odours and alarm cues consistent with detecting elevated risk levels. In contrast, Nile tilapia embryos only demonstrated significant risk responses to embryonic alarm cues. These findings indicate that embryos of both species can innately recognize alarm cues but differ in their response patterns to conspecific odours. The results highlight that parental care strategies may influence intergenerational chemical communication in fish embryos.

## Simple Summary

Chemical odours are vital for aquatic organisms’ communication, yet whether fish embryos are innately capable of responding to ecologically relevant chemical cues released by cohort competitors, potential caring or risky cues released by parents, or chemical alarm cues released by damaged embryos, and whether response patterns differ between species employing distinct parental care strategies, remain unclear. Our research reveals that reproductive strategy may influence intergenerational chemical communication in fish embryos, with maternal odours perceived as relatively risky when parental care is absent but not when parental care is present within species. This study is the first to explore the recognition and response of embryos to environmental chemical cues based on the differences in parental care.

## 1. Introduction

Chemical communication is a widespread phenomenon that plays crucial roles in intra- and interspecific interactions across various biological communities [1,2,3,4]. From bacteria and fungi to plants and animals, organisms have developed mechanisms to communicate through the release and/or detection of chemical signals [2,5,6,7,8]. Chemical communication enables organisms to interact with and respond to their environment, coordinate social behaviours, and convey and receive important cues related to reproduction, predation, territoriality, and resource availability [9,10,11,12,13].

Aquatic animals, including fishes, rely greatly on chemical cues [14,15,16] for survival and environmental adaptation [17,18] as visual information is often limited due to factors including water turbidity and light attenuation with depth [3,19]. Nonetheless, fish may exhibit plasticity in their chemical communication patterns that are influenced by balancing trade-offs between ecological costs and benefits [20]. Notably, prey fishes often face a conflict between foraging requirements and avoiding predation [15,21,22], as foraging activities that maximize food intake may increase their detectability and vulnerability to predators [23,24,25]. To optimize this trade-off, prey fish should adjust their behavioural responses based on the perceived degree of contemporaneous predation risk [13,26,27].

Describing the response patterns of different fishes to environmental chemical cues in critical early life history stages may provide an empirical basis to inform the development and optimization of fish conservation initiatives in the context of environmental change, including artificial breeding and captive rearing for conservation stocking programs. Although a large number of studies have explored behavioural and physiological responses to a diverse array of non-lethal, environmentally-derived chemical cues in post-larval fishes, embryonic responses are poorly represented in the literature. It remains unclear whether fish embryos are innately responsive to ecologically relevant chemical cues, especially to cues from damaged conspecific embryos that could be indicative of predation, or to the odours of cohort competitors. As many captive breeding and stocking programs involve artificial fertilization and incubation free from parental influence, innate responses to potential care-associated or risk-indicating cues from parents may be informative from an applied perspective, particularly if patterns of response to parental cues vary with parental care strategies, i.e., from low to high levels of parental care under natural conditions.

Fish have developed a diverse array of reproductive strategies, ranging from broadcast spawning and scattering eggs in the environment to internal fertilizations and true viviparity [28,29,30,31]. In some species, parental care ostensibly promotes reproductive success through enhanced offspring survival [32]. Conversely, in species without parental care and where opportunistic cannibalism by adults may occur [33], maternal odours may effectively serve as risky cues, warning developing embryos of elevated predation risk. As it is unclear whether embryonic fishes in general possess innate recognition of chemical alarm cues from damaged conspecifics, we propose a hypothesis wherein innate alarm cue responses in fish embryos may differ between species with different parental care strategies, such that high degrees of parental care may be associated with lower levels of response if parental care generally reduces predation risk (Hypothesis 1). During the early stages of development, embryos compete for limited resources such as oxygen to ensure individual survival [34]. Little attention has been paid to the potential effects of sibling embryo cues on behavioural responses and developmental processes. Considering the competitive relationships among multiple individuals at the high densities often associated with embryonic development, we propose a second hypothesis that companion (sibling) chemical cues accelerate embryonic development of broodmates (Hypothesis 2). Finally, considering the range of potential effects of parental chemical cues on embryos, we propose a hypothesis wherein intergenerational chemical communication from parents to embryos varies between parental care strategies with a negative relationship between degree of parental care provided and risk content of chemical cues (Hypothesis 3).

To test these three hypotheses, we evaluated the responses of embryos including heart rate and incubation performance to different conspecific chemical cues (water control, companion embryo odours, maternal odours, maternal + companion odours, and embryonic damage-released chemical alarm cues) in a species without parental care (zebrafish, *Danio rerio*) and a species with parental care (Nile tilapia, *Oreochromis niloticus*). Zebrafish is a widely used model species with a tendency to consume their own embryos [35,36,37]. Nile tilapia is an important aquaculture species recommended by the UN FAO to help address food scarcity issues [38] but which has achieved biologically invasive status in many countries and regions. Tilapia are well-known for their parental care behaviour, with the mother mouth-brooding her embryos until hatching [39,40].

## 2. Methods

### 2.1. Test Subjects

Zebrafish and Nile tilapia used in this study were obtained from established laboratory populations and were maintained according to standard culture protocols [41]. The founding strain of Nile tilapia was originally from Egypt and provided by Prof. Y. Nagahama (Laboratory of Reproductive Biology, National Institute for Basic Biology, Okazaki, Japan). Aquaria housing zebrafish (40 cm length × 23 cm width × 42 cm height, filled to 26 cm depth) and Nile tilapia (65 cm length × 65 cm width × 45 cm height, filled to 40 cm depth) were filled with dechlorinated water with dissolved oxygen levels greater than 6 mg/L on a 14: 10 h light–dark cycle. The males and females of both species were kept separately in different tanks with water temperature maintained at 24 ± 0.5 °C for zebrafish and 27 ± 0.5 °C for Nile tilapia, respectively. Zebrafish were fed ad libitum twice daily with frozen blood worm (*Tubifex* spp.), and Nile tilapia were fed twice daily with commercial fish feed (composition: crude protein ≥ 30%, crude fat ≥ 3.0%, fiber ≤ 8.0%, ash ≤ 16%, and lysine ≥ 1.2%).

### 2.2. Reproduction of Zebrafish and Nile Tilapia

Zebrafish (one male and one female) were placed in a custom breeding box (14 cm length × 8 cm width × 10 cm height, filled to 7 cm depth) with an opaque cover at 21:00, one hour before lights-off, and the temperature was maintained at 24 ± 0.5 °C. Lights were switched on the following morning at 08:00 and the resulting embryos were collected one hour later. To reduce potential maternal differences, only females with similar fecundity (300–350 eggs per batch) and good embryo quality (assessed visually) after spawning were selected for the experimental groups. A total of 17 males and 17 females and their embryos were used in this experiment.

Nile tilapia (one male and one female) were carefully selected for artificial fertilization. The fertilized embryos were then transferred to a circulating water incubation system, where the water pump flow rate was adjusted to ensure a moderate flow in the incubation tubes. To eliminate potential maternal differences, only females with similar fecundity (1500–2000 eggs per batch) and good embryo quality (assessed visually) after spawning were chosen for the experimental groups. A total of 17 males and 17 females and their embryos were used in this experiment.

### 2.3. Cultivation of Embryos

Embryo cultivation was performed in cell culture plates, and to exclude possible interference from other chemical cues, each embryo to be tested was cultivated individually, with 1 embryo per well of each culture plate. Based on the relative size of the embryo volumes, zebrafish and tilapia embryos were incubated with a concentration of 1 embryo/mL of water and 1 embryo/3 mL of water, respectively. To simulate the natural incubation process of Nile tilapia, the plates containing embryos were placed in a horizontal shaker (TYZD-IIIA, Shanghai, China) with a rotational speed of 60–70 r/min. The conditions of temperature, photoperiod, and dissolved oxygen during cue preparation were consistent with the parental rearing environment, and half of the water volume was changed in each culture plate daily.

### 2.4. Treatment Preparation

To prepare companion embryo odours (treatment CEO), *n* = 100 zebrafish embryos and *n* = 60 Nile tilapia embryos, originating from the same mother as the experimental embryos, were collected. Following a ratio of 1 embryo/mL water, the zebrafish embryos were placed in a beaker containing 100 mL of aerated water, and then the beaker was put into a thermostatic water bath (DKS-14, Chongqing, China) at a temperature of 24 ± 0.5 °C. With a ratio of 1 embryo/3 mL water, the Nile tilapia embryos were placed in a beaker containing 180 mL of aerated water, and then the beaker was placed in a horizontal shaker with a rotational speed of 60–70 r/min at a temperature of 27 ± 0.5 °C. The conditions of temperature, photoperiod, and dissolved oxygen during cue preparation were consistent with the parental rearing environment, and half of the water volume in each beaker was changed daily. The fraction of the soaking solution that was replaced each day as described above was then filtered through a 74 μm (200-mesh) sieve to obtain companion embryo odours from donors at the developmental state of the experimental embryos to be tested.

To prepare maternal odours (treatment MO), subsets of the maternal zebrafish (heart rate measurement group: 0.71 ± 0.07 g, 3.38 ± 0.06 cm, *n* = 4; incubation performance measurement group: 0.71 ± 0.02 g, 3.36 ± 0.04 cm, *n* = 13) and maternal Nile tilapia (heart rate measurement group: 353.66 ± 62.58 g, 22.13 ± 1.28 cm, *n* = 4; incubation performance measurement group: 364.63 ± 41.70 g, 22.75 ± 0.41 cm, *n* = 4) were used. Based on preliminary experimental studies, maternal fish were immersed in fresh water at a ratio of 1 g/500 mL water and fasted for 24 h after spawning. The temperature, light cycle, dissolved oxygen, and other environmental conditions were maintained consistent with those of the parental fish holding tanks. The soaking solutions of maternal odours were filtered as above but packaged in 20 mL aliquots stored at −20 °C until use. For the maternal + companion embryo odours (treatment CEO + MO), companion embryo odour solution and maternal cue solution were blended at equal (1:1) concentrations for each species before freezing.

Embryonic alarm cues (EAC) were generated for each sibling group and collected from embryos sharing the same mother as the cue recipient. Zebrafish and tilapia embryos were crushed and ground at concentrations of 2 embryos/mL water and 2 embryos/3 mL water, respectively. The crushed embryos were then centrifuged at 1000 r/min for 5 min (5920R centrifuge, Eppendorf, Germany) at 4 °C, and the supernatant was packaged and stored as above.

### 2.5. Determination of Embryo Heart Rate in Zebrafish

Once the zebrafish embryos reached the pharyngula stage (showing pigment in the middle region of the back but not yet across the entire body), 140 embryos were selected for heart rate measurements to examine their responses to the different treatments (water control, CEO, MO, CEO + MO, and EAC; *n* = 28 each, *n* = 140 total). The pharyngula stage was selected as it represents the earliest point in development when the olfactory system is likely functional, enabling the embryo to perceive chemical information. Morphologically, this stage was easily identifiable, ensuring consistency in the developmental status of the test embryos.

Slides with grooves (planar aperture: 16 mm, aperture depth: 2 mm) were placed on the platform of a fluorescence body microscope (M205 FCA, Leica, Germany). First, individual embryos and 200 μL of culture medium were transferred into the grooves via pipette. Embryonic heart rate was measured (pre-test) by video recording for 30 s after a 2 min acclimation period. Second, the embryo and 200 μL of culture medium were transferred to a new cell culture plate; 800 μL of treatment solution was added and maintained for a 2 min treatment time. Third, the embryo and 200 μL of mixed fluid (embryo culture medium + treatment) were transferred with a pipette into the groove of the slide, and the embryonic heart rate was measured (post-test) over an additional 30 s video recording after a 2 min acclimation period. The changes in embryonic heart rate (post-test minus pre-test) following exposure to the different treatments were then analyzed. To minimize observer bias, video data were scored without reference to treatment.

### 2.6. Determination of Embryo Hatching Performance in Zebrafish

During the initial 24 h of incubation, the embryos were individually cultivated in water, after which the culture medium was replaced with an equal volume of one of the treatment solutions via transfer pipette. Half of the volume was then replaced daily with the appropriate treatment until the embryos completed incubation. The conditions of temperature, photoperiod, and dissolved oxygen during hatching were consistent with the parental rearing and egg fertilization environments.

Initial hatching time was recorded when the first embryo hatched, and subsequently, the number of hatched embryos, dead embryos, and deformed embryos was recorded every 0.5 h until the last viable embryo hatched. After incubation, the hatching rate, deformation rate, and embryo mortality, as well as the final hatching time, membrane rupture duration (i.e., the time between initial and end hatching), and hatching duration (time taken for 50% of the embryos to hatch), were analyzed. To analyze embryo hatching performance, 16 embryos were considered one observation unit, and the sample size for all treatment groups was *n* = 13 (*n* = 1040 embryos total).

### 2.7. Determination of Embryo Heart Rate and Hatching Performance in Nile Tilapia

Once the Nile tilapia embryos reached the pharyngula stage (showing pigment in the middle region of the back but not yet across the entire body), 100 embryos were selected for heart rate measurements to examine their responses to the five different treatments (water control, CEO, MO, CEO + MO, and EAC; *n* = 20 each, *n* = 100 total). Heart rate was determined for tilapia as described above for zebrafish.

Hatching performance was determined for tilapia in the same way as for zebrafish as described above, with 18 embryos considered as one observation unit, and the sample size for all treatment groups was *n* = 9 (*n* = 810 embryos total).

### 2.8. Statistical Analysis

Blind methods were employed throughout the recording and analysis of all behavioural data to minimize observer bias. Probabilistic regression of hatching rate over time for individual samples in each treatment group was performed to calculate the incubation duration of the embryos using the Bliss method. If the response variables met the assumptions of normality and homogeneity of variances, one-way ANOVAs were used to test for differences among the treatment groups. If the assumptions were not met, the Kruskal–Wallis and Nemenyi’s post hoc (with Tukey distribution) tests were used to analyze differences between treatments in embryo heart rate and incubation performance measures. Hatchability curves against incubation time were generated from generalized additive mixed models (GAMMs) using the packages *mgcv* [42] and *nlme* [43]. All of these analyses were carried out in R v3.4 (R Core Team 2020).

## 3. Results

### 3.1. Zebrafish Embryonic Heart Rate Responses to Chemical Cues

**Figure 1:**
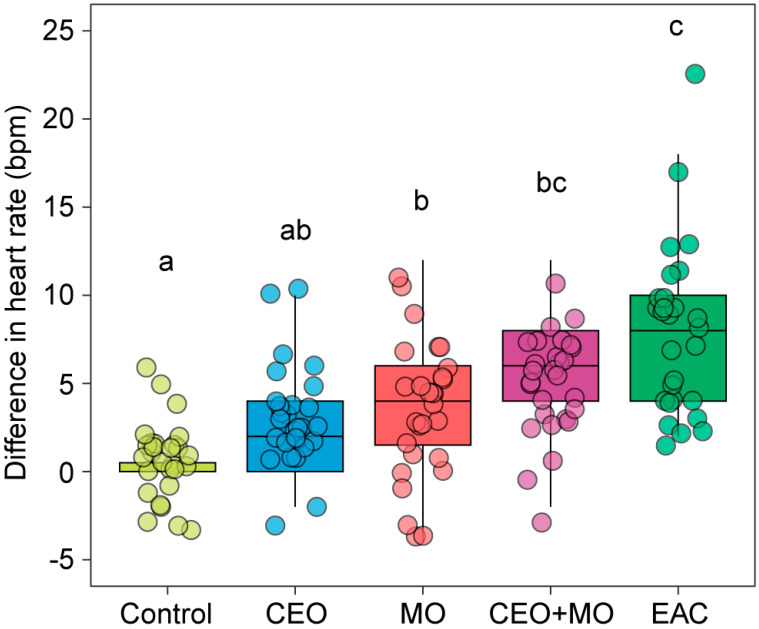
Heart rate (bpm) differences from the pre- to post- stimulus observation periods of zebrafish in response to treatments. Different letters denote statistically significant differences (*p* < 0.05) between treatments from Nemenyi’s post hoc test. Control: water; CEO: companion embryo odours; MO: maternal odours; CEO + MO: maternal + companion embryo odours; EAC: embryonic alarm cues.

There were statistically significant differences in the change in zebrafish embryonic heart rate in response to treatment (*p* < 0.0001; Table 1). Each treatment elicited significantly higher heart rates in zebrafish embryos than the control group (all *p* < 0.05) with the exception of the companion embryo odours (Figure 1). There were stepwise increases in magnitudes of response with control < CEO < MO < CEO + MO < EAC, with EAC responses significantly greater than all but CEO + MO (Figure 1).

### 3.2. Zebrafish Hatching Performance Responses to Chemical Cues

**Figure 2:**
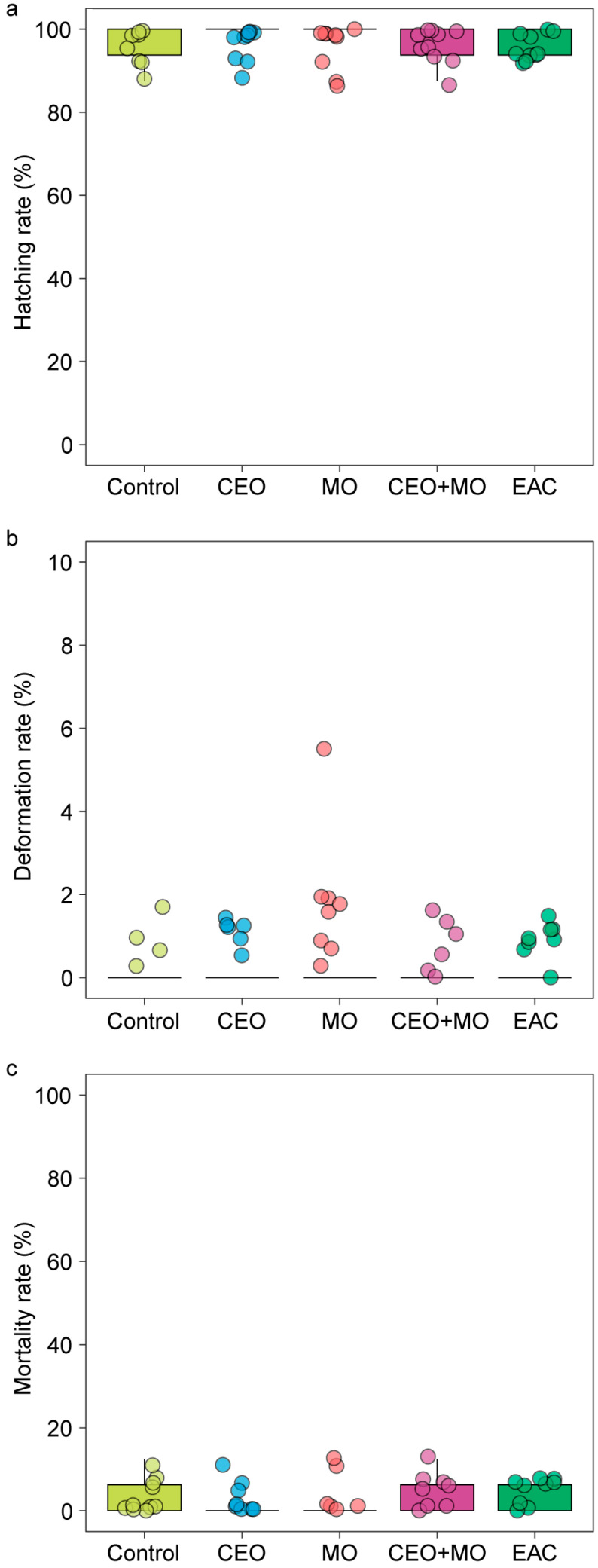
The (**a**) hatching rate, (**b**) deformation rate, and (**c**) overall mortality of zebrafish embryos in response to treatments. Control: water; CEO: companion embryo odours; MO: maternal odours; CEO + MO: maternal + companion embryo odours; EAC: embryonic alarm cues.

**Figure 3:**
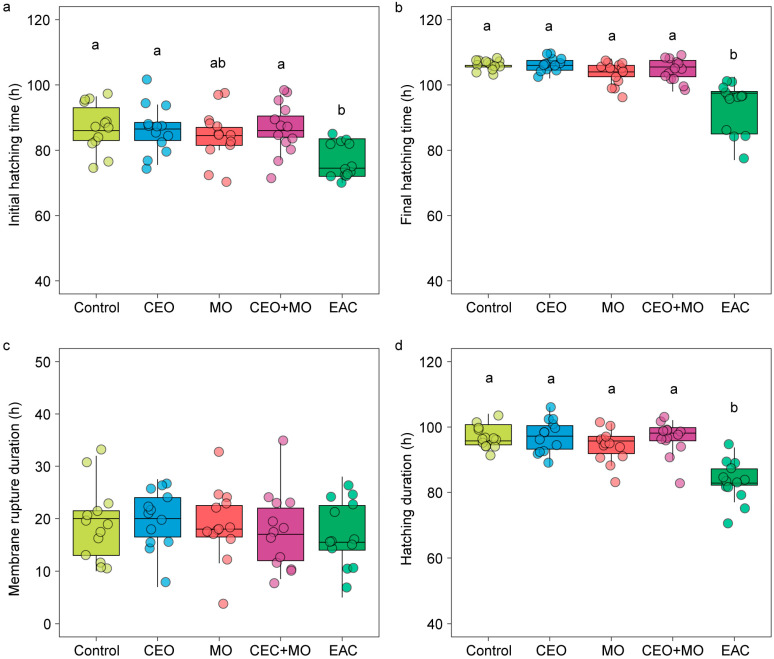
The (**a**) initial hatching time, (**b**) final hatching time, (**c**) membrane rupture duration, and (**d**) hatching duration of zebrafish embryos in response to treatments. Different letters denote statistically significant differences (*p* < 0.05) between treatments from Nemenyi’s post hoc test. Control: water; CEO: companion embryo odours; MO: maternal odours; CEO + MO: maternal + companion embryo odours; EAC: embryonic alarm cues.

**Figure 4:**
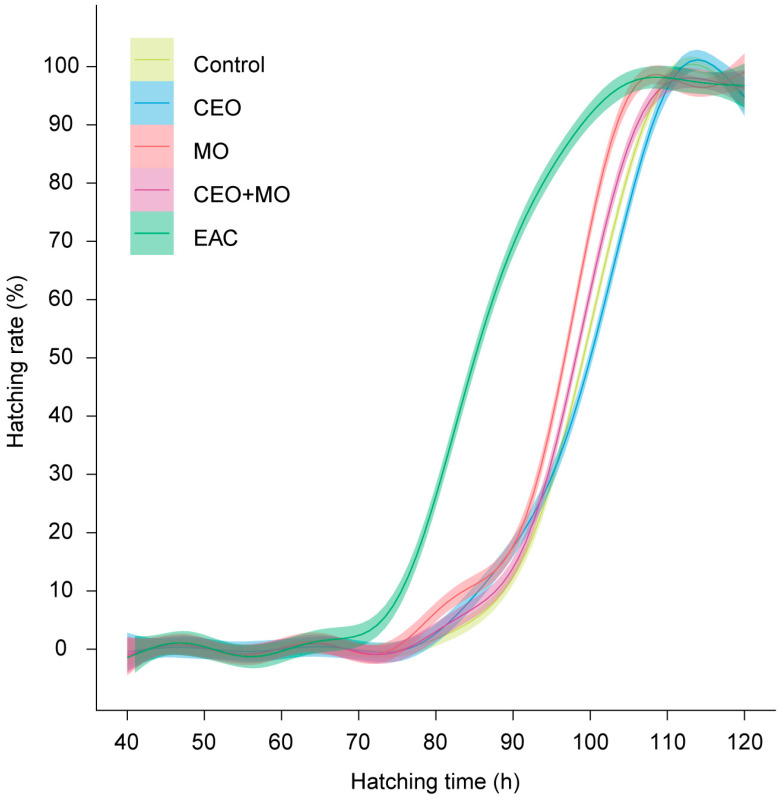
Hatchability curves of zebrafish embryos against incubation time under different treatments. Control: water; CEO: companion embryo odours; MO: maternal odours; CEO + MO: maternal + companion embryo odours; EAC: embryonic alarm cues.

There was no significant difference in hatching rate (*p* = 0.841; Figure 2a), deformation rate (*p* = 0.406; Figure 2b), or mortality (*p* = 0.693; Figure 2c) of zebrafish embryos in response to treatment (Table 1). However, treatment had a significant effect on the initial hatching time (*p* = 0.002) and the final hatching time (*p* < 0.0001; Table 1). Specifically, embryonic alarm cue (EAC) resulted in significantly shorter initial hatching time (Figure 3a) and shorter final hatching time (Figure 3b) compared to the other treatments. There was no significant difference in membrane rupture duration (*p* = 0.853; Figure 3c) but there was a significant difference in hatching duration (*p* < 0.0001; Table 1), where EAC resulted in significantly shorter hatching duration than all other treatments (*p* < 0.05), which did not differ from each other in any pairwise comparison (all *p* > 0.05; Figure 3d). Hatchability curves of zebrafish embryos against incubation time were similar between the control and odour treatments, while alarm cues were associated with faster hatch times (Figure 4).

### 3.3. Nile Tilapia Embryonic Heart Rate Responses to Chemical Cues

**Figure 5:**
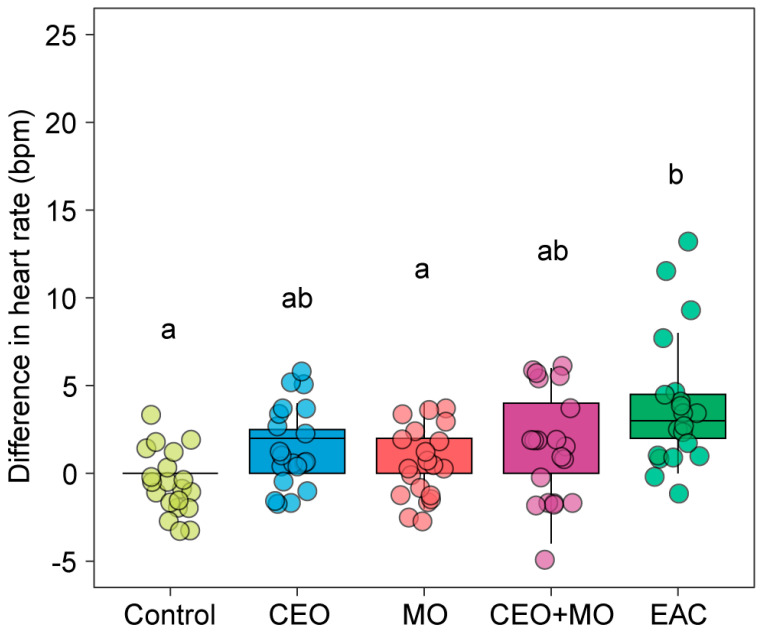
Heart rate (bpm) differences from the pre- to post- stimulus observation periods of Nile tilapia in response to treatments. Different letters denote statistically significant differences (*p* < 0.05) between treatments from Nemenyi’s post hoc test. Control: water; CEO: companion embryo odours; MO: maternal odours; CEO + MO: maternal + companion embryo odours; EAC: embryonic alarm cues.

There were significant effects of treatment on the change in Nile tilapia embryonic heart rates (*p* < 0.0001; Table 1). However, only embryonic alarm cues (EAC) resulted in a significant increase in embryo heart rate compared to the control (*p* < 0.0001), with the odour treatments showing no significant difference compared to the control (all *p* > 0.05). There was no evidence of any patterning of responses between the non-control treatments, with alarm cues differing significantly from maternal odours (MO) but not companion embryo odours (CEO) or the maternal + companion odour (CEO + MO) combination (Figure 5).

### 3.4. Nile Tilapia Hatching Performance Responses to Chemical Cues

**Figure 6:**
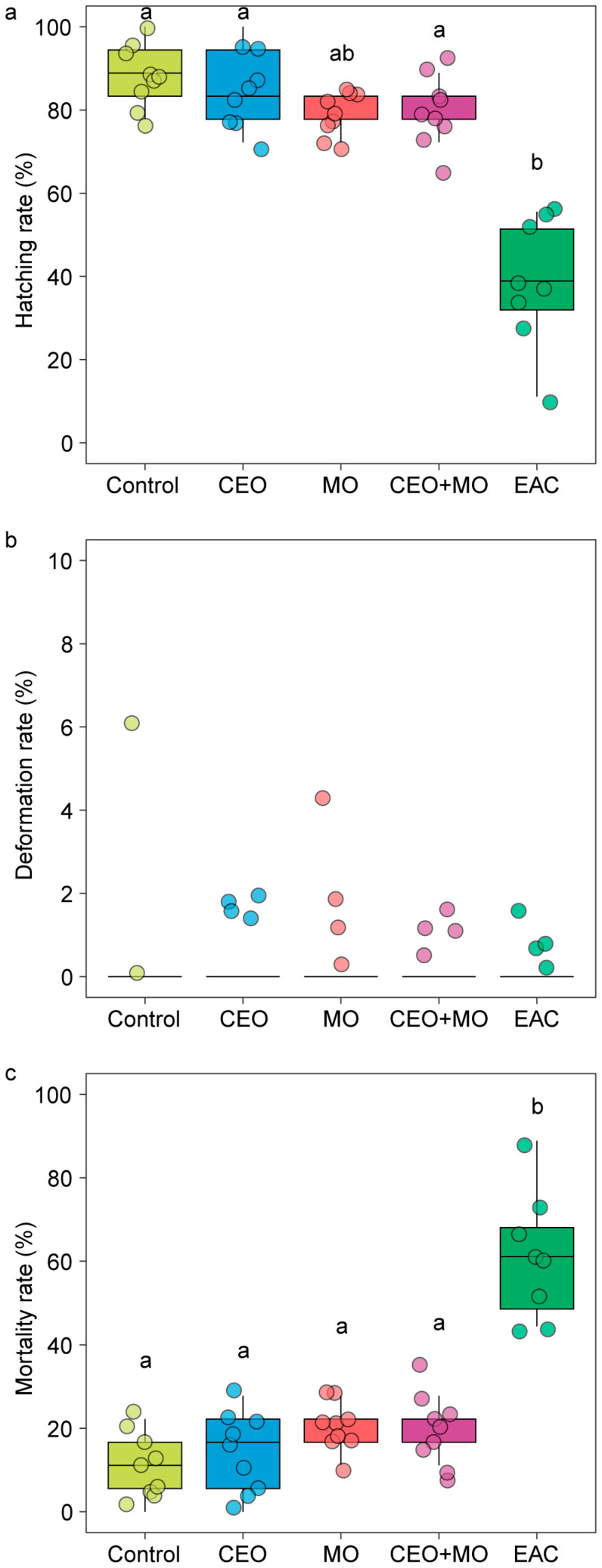
The (**a**) hatching rate, (**b**) deformation rate, and (**c**) overall mortality of Nile tilapia embryos in response to treatments. Different letters denote statistically significant differences (*p* < 0.05) between treatments from Nemenyi’s post hoc test. Control: water; CEO: companion embryo odours; MO: maternal odours; CEO + MO: maternal + companion embryo odours; EAC: embryonic alarm cues.

**Figure 7:**
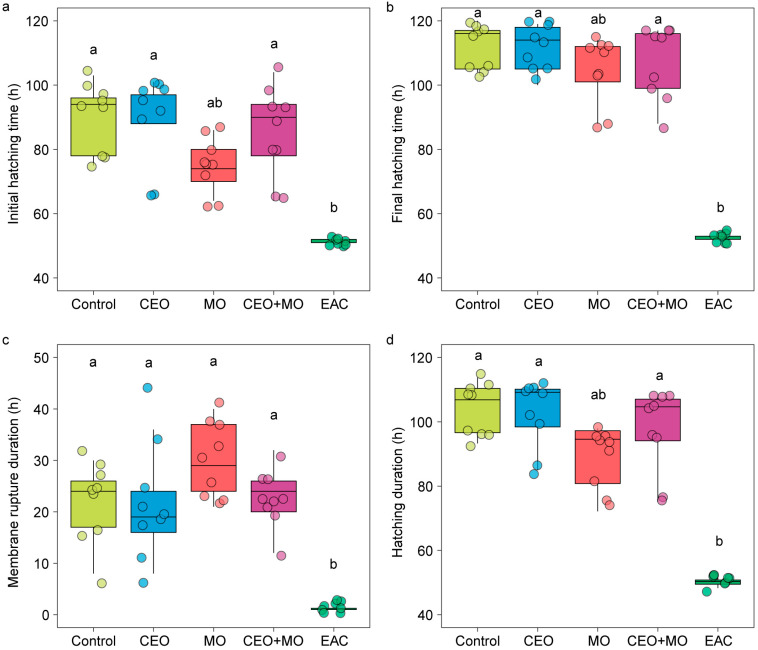
The (**a**) initial hatching time, (**b**) final hatching time, (**c**) membrane rupture duration, and (**d**) hatching duration of Nile tilapia embryos in response to treatments. Different letters denote statistically significant differences (*p* < 0.05) between treatments from Nemenyi’s post hoc test. Control: water; CEO: companion embryo odours; MO: maternal odours; CEO + MO: maternal + companion embryo odours; EAC: embryonic alarm cues.

**Figure 8:**
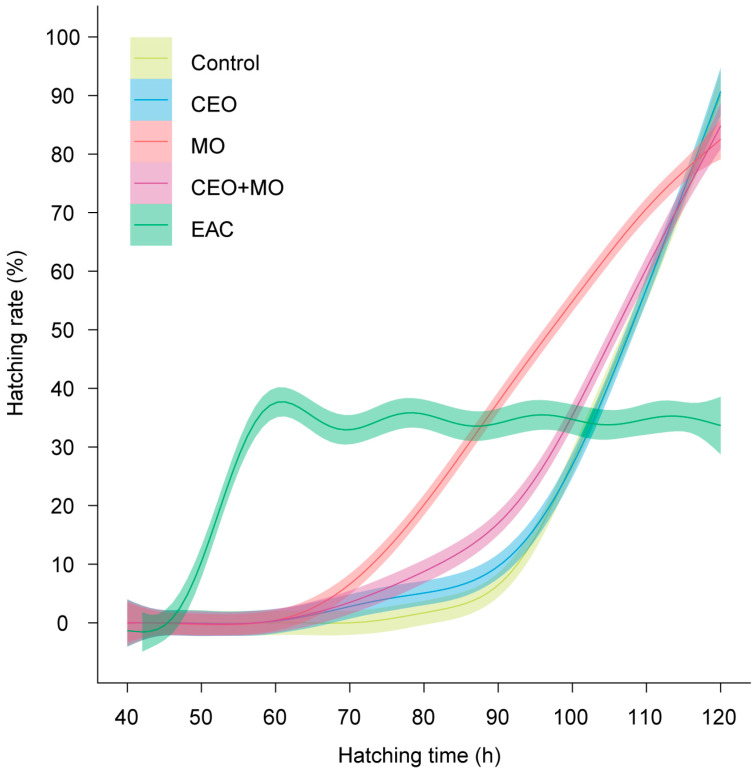
Hatchability curves of Nile tilapia embryos against incubation time under different treatments. Control: water; CEO: companion embryo odours; MO: maternal odours; CEO + MO: maternal + companion embryo odours; EAC: embryonic alarm cues.

Treatment had a significant effect on the hatching (*p* < 0.0001; Figure 6a) and mortality rates (*p* < 0.0001; Figure 6c) of Nile tilapia embryos, with embryonic alarm cues (EAC) resulting in significantly lower hatching and higher mortality rates than all other treatments (*p* < 0.05) except for the maternal odours (MO)—EAC comparison in hatch rate (Figure 6a). All other treatments did not differ from each other in any pairwise comparison (all *p* > 0.05; Figure 6a,c). Treatment had no effect on the deformation rate (*p* = 0.546; Figure 6b) but had a significant effect on the initial (*p* < 0.0001; Figure 7a) and final hatching times (*p* < 0.001; Figure 7b, Table 1). EAC resulted in significantly shorter initial and final hatching times (both *p* < 0.05) than all other treatments except for the maternal odours, while no significant differences were observed in any other pairwise comparison (all *p* > 0.05). Treatment also had significant effects on membrane rupture duration (*p* < 0.001; Figure 7c) and hatching duration (*p* < 0.0001; Figure 7d, Table 1), with EAC associated with significantly shorter membrane rupture and hatching durations. Overall, the hatchability curves of Nile tilapia embryos were notably different between treatments, with the control and companion embryo odours (CEO) treatments showing the slowest hatch rates, MO and CEO + MO treatments demonstrating intermediate hatch rates, and EAC treatment demonstrating significantly faster initial hatch rates but a lower overall hatch success of ~40% compared to success >80% in the other four treatments (Figure 8).

## 4. Discussion

In this study, embryonic zebrafish and Nile tilapia demonstrated responses to conspecific chemical alarm cues consistent with antipredator strategies. In the short term (minutes), both species demonstrated significant post-stimulus increases in heart rate compared to control treatments. Over longer periods (days), alarm cue-exposed embryos demonstrated significantly shorter hatch times indicating altered developmental trajectories. This early hatching did not result in decreased hatch success for zebrafish but was associated with a significant reduction in hatch success in tilapia, dropping to ~40% from the ~80–90% observed in the other four treatment groups. Moreover, the response patterns to conspecific odours varied inconsistently, with maternal odours associated with weakly elevated heart rate levels in zebrafish but not in tilapia. Pairing maternal and embryo odours did not elicit responses different to either odour in isolation in zebrafish but tended to elicit responses more similar to those of embryo odours than to maternal odours in tilapia. In general, the observed response patterns in embryos reflect interspecific differences in parental care strategies and associated risk of cannibalism.

Fishes are generally considered to have the ability to innately recognize chemical cues from conspecifics and discriminate between kin and non-kin [44,45,46,47]. The responses we observed to alarm cues supported our first hypothesis, that embryos of these species do possess innate responses to some conspecific chemical cues, including alarm cues. Fish embryos can exhibit shortened incubation periods and precocious maturation as a response to elevated environmental risks [48], and chemical alarm cues from conspecific larvae or adult fish have previously been shown to accelerate the incubation process in zebrafish embryos [49,50]. This accelerated incubation can have consequences for the phenotype of the offspring, such as smaller body size and increased vulnerability to predation at hatching [48]. Our results support these findings by demonstrating that exposure to chemical alarm cues led to increased heart rate and shorter incubation in both zebrafish [48,49] and Nile tilapia embryos. While chemical alarm cues significantly shortened the hatching duration of Nile tilapia embryos, they also concurrently resulted in high embryonic mortality. Notably, most of the dead embryos were underdeveloped, with unabsorbed yolk sacs, poorly defined body segments, and incompletely formed circulatory systems. The findings indicate that the clearance of damaged and dead embryos might constitute a critical component of tilapia parental care. From a chemical communication perspective, removing impaired embryos is essential for improving hatching quality in certain fish within aquaculture production. On the other hand, chemical alarm cues elicited extremely short membrane rupture duration times in Nile tilapia embryos, indicating a surprisingly synchronized response to high perceived levels of risk in the environment. However, alarm cue exposure had no apparent effect on survival of zebrafish embryos. This may suggest that zebrafish embryos have a greater ability to cope with environmental risks, possibly due to the absence of risk-mediating parental care behaviour. In contrast, Nile tilapia are incubated in the mouth of the mother fish from the embryo stage, and even newly hatched fry will be taken back into the mouth of the mother to avoid predation risk. 

The ability to recognize kin may reduce the risk of inbreeding and facilitate group formations that decrease predation risk to individuals through dilution [51,52]. However, competition for oxygen and other resources among companion embryos can occur at high densities, which can lead to reduced larval survival rates and potentially influence population-level processes [53,54]. High density has been shown to have adverse effects on the survival rates of three-spined stickleback (*Gasterosteus aculeatus*) embryos, which can ultimately limit adult recruitment and population size [55]. In this study, both zebrafish and Nile tilapia embryos did not demonstrate significant responses to the odours of companion embryos, although these tended to result in elevated embryonic heart rates compared to control groups in both species. Our results did not support our second hypothesis that companion embryo cues accelerate embryonic development. Possible explanations for this include the following: (1) the concentration or quantity of the companion embryo cues used in this study was insufficient to elicit a noticeable sensitivity response in the embryos; (2) the density of companion embryos in this study was not sufficient to induce significant competitive effects among the embryos of either fish species, hence having no impact on heart rate and incubation performance; (3) isolated cultivation deprived embryos of developmental social environment, weakening their response to companion embryo odours in competitive scenarios; or (4) competition during embryonic development in the experimental fish was primarily direct and resource-utilizing rather than chemical cue-mediated indirect interference.

Maternal odours and embryonic alarm cues had similar patterns of effect in the parental care-lacking zebrafish embryos, with both treatments were associated with noteworthy increases in heart rates compared to the control treatment. This suggests that maternal odours in zebrafish may convey information on ambient risk similar to that conveyed by chemical alarm cues. On the other hand, alarm cues but not maternal odours were also associated with shorter initial and final hatching times, as well as faster overall hatch rates, which may indicate that these two types of cues differ in the degree of risk they represent. Maternal odours had no significant effects on the heart rate or hatching performance of Nile tilapia while alarm cues were associated with increased heart rates, decreased hatch rates, higher embryo mortality, shorter hatch times, and shorter durations that imposed severe survival costs. Although Nile tilapia exhibit relatively high levels of parental care behaviour toward their offspring compared to zebrafish, they may also consume their own embryos in certain circumstances to supplement their diet consistent with facultative or opportunistic kin cannibalism. The potential components of maternal odours include steroids, prostaglandins, amino acids, and metabolic byproducts from skin mucus and secretions, forming a complex array of chemical cues [56,57]. Here, we observed non-significant differences in response to maternal odours versus controls, but whether these suggest maternal odours as conveying non-zero levels of risk or simply a generalized stimulus is unresolved. In general, these findings partially support our third hypothesis, that parental care strategies may influence intergenerational chemical communication and risk detection in fish embryos due to the risk-mediating effects of parental care itself.

Reproductive strategies are highly conserved within families and genera [58]. Parental care putatively enhances offspring survival in teleosts, and approximately 25% of all fish families exhibit some degree of parental care [31]. Notably, although some fishes exhibit parental care behaviour, opportunistic cannibalism can sometimes occur in species that demonstrate parental care [59]. For example, in a cichlid (*Pseudocrenilabrus multicolor*) in which the female incubates fertilized eggs in her mouth, selective consumption of unfertilized eggs has been observed [60]. Under conditions of extreme resource limitation, mouth-brooding parents may also consume some of their fertilized eggs [60,61]. Occasionally, if the number of remaining eggs in a mouth-brooded clutch drops below 20% of the total spawn, incubation may be abandoned and the remaining eggs consumed [62,63]. In terms of evolutionary adaptation, Nile tilapia may have not developed similarly advanced mechanisms for coping with risks during early life stages as zebrafish. Additionally, the higher hatching rates and lower mortality rates observed in both maternal odour, and maternal + companion odours groups of Nile tilapia embryos are consistent with the notion that maternal fish may promote the survival and hatching rates of viable embryos by promptly eliminating damaged or dead embryos, possibly through selective consumption [64]. Differences in response to conspecific odours may reflect differences in life history patterns if high degrees of parental care such as mouth brooding promote growth and survival across embryonic progeny and limit faster growth and development of precocious individuals, decreasing the risk of intra-cohort cannibalism.

## 5. Conclusions

Both zebrafish and Nile tilapia embryos demonstrated the ability to recognize conspecific chemical alarm cues in their environment through physiological and developmental responses, and alarm cue exposure resulted in significant mortality in tilapia. Their response patterns to conspecific odours vary, consistent with predictions based on differences in parental care strategies, with maternal odours associated with elevated heart rate levels in the parental care-lacking zebrafish but not in tilapia. This study is the first to explore the recognition and response of embryos to environmental chemical cues based on differences in parental care strategies. Our findings provide experimental context for theoretical studies of chemical communication and parental care in the early life-history stage of fishes.

## Figures and Tables

**Table 1:** Effects of treatments on embryo performance of zebrafish and Nile tilapia.

	Embryonic Heart Rate	Hatching Performance						
Species	Difference in Heart Rate	Hatching Rate	Deformation Rate	Mortality	Initial Hatching Time	Final Hatching Time	Membrane Rupture Duration	Hatching Duration
Zebrafish	*H*_4_ = 53.621 *p* < 0.0001	*H*_4_ = 1.420 *p* = 0.841	*H*_4_ = 4.000 *p* = 0.406	*H*_4_ = 2.232 *p* = 0.693	*H*_4_ = 16.953 *p* = 0.002	*H*_4_ = 30.426 *p* < 0.0001	*F*_4,65_ = 0.335 *p* = 0.853	*H*_4_ = 26.865 *p* < 0.0001
Nile tilapia	*H*_4_ = 24.063 *p* < 0.0001	*H*_4_ = 26.171 *p* < 0.0001	*H*_4_ = 3.070 *p* = 0.546	*H*_4_ = 25.954 *p* < 0.0001	*H*_4_ = 24.916 *p* < 0.0001	*H*_4_ = 22.225 *p* < 0.001	*F*_4,44_ = 23.472 *p* < 0.001	*H*_4_ = 25.935 *p* < 0.0001

*H*-statistics are from Kruskal–Wallis tests, *F*-statistics are from one-way ANOVA.

## Data Availability

Data are freely available from the Open Science Framework (https://osf.io/7xkav/; doi: 10.17605/OSF.IO/7XKAV, accessed on 5 February 2025).
